# P-478. Assessing HIV Transmitted Drug Resistance and cluster detection among Antiretroviral-Naïve individuals in Hispaniola

**DOI:** 10.1093/ofid/ofae631.677

**Published:** 2025-01-29

**Authors:** Hector J Lora, Martha Sanchez, Vladimir Novitsky, Joel Hague, Meghan McCarthy, Allison DeLong, Diego Mendoza, Ingrid Ruiz, Maridania Jabier, Robert Paulino-Ramírez, Rami Kantor

**Affiliations:** Instituto de Medicina Tropical y Salud Global (IMTSAG), Santo Domingo, Distrito Nacional, Dominican Republic; Brown University, Providence, Rhode Island; Alpert Medical School of Brown University, Providence, Rhode Island; Brown University, Providence, Rhode Island; Brown University, Providence, Rhode Island; Brown University, Providence, Rhode Island; UNIBE, Santo Domingo, Distrito Nacional, Dominican Republic; UNIBE, Santo Domingo, Distrito Nacional, Dominican Republic; UNIBE, Santo Domingo, Distrito Nacional, Dominican Republic; Universidad Iberoamericana, Santo Domingo, Distrito Nacional, Dominican Republic; Alpert Medical School of Brown University, Providence, Rhode Island

## Abstract

**Background:**

The use of antiretroviral therapy (ART) has surged globally, heralding an era of life-saving treatment for millions. However, with this widespread adoption of ART, a concerning trend has emerged: the escalation of antiretroviral resistance. Within the confines of La Hispaniola, encompassing the Dominican Republic (DO) and Haiti(HT), the prevalence of HIV stands among the highest in the Caribbean region. Our main objective is to identify and characterize the extent of transmitted drug resistance (TDR) of HIV among treatment naïve participants, its demographic and the clinical associations in the DO and the transmission networks.

Phylogenetic relationships between DRII PRRT sequences and NNRTI SDRMs

**Figure:**
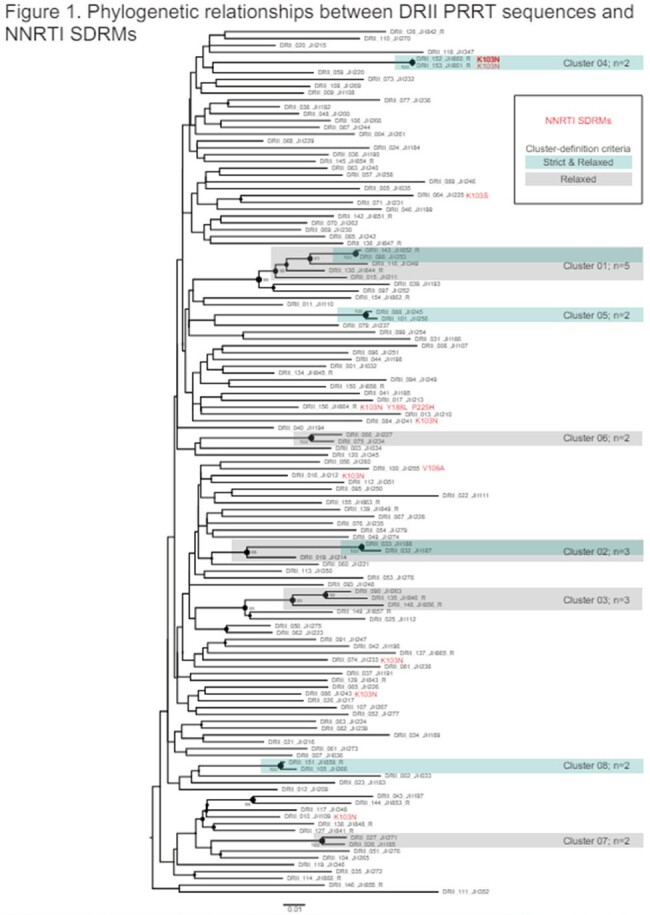

**Methods:**

During May 2021-June 2022, we enrolled 148 newly HIV-diagnosed ART naïve adults at Centro de Orientacion e Investigación (COIN) in DO. Demographic and clinical were collected, and blood samples obtained for partial *pol* genotyping using NGS. Subtyping and resistance interpretation were performed with Stanford Database tools. Maximum likelihood phylogenies were inferred by RAxML and clusters were defined as clades with bootstrap support of ≥0.8. Extended phylogenetic analyses included ∼1,500 publicly available Caribbean sequences including prior data set from our group. Fisher Exact tests and logistic regression were used to test associations of those in clusters.

Phylogenetic relationships of DRI and DRII PRRT sequences

**Figure:**
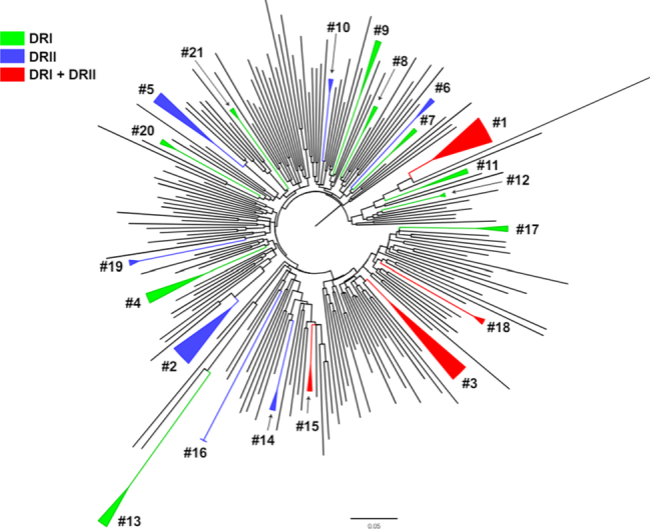

**Results:**

Among participants the mean age was 30 years, 52% male, 48% were Dominican and 52% Haitian, 74% heterosexual. Among those with genotype, 8% (10 out of 127) had NNRTI SDRMs (Figure 1). A minority SDRMs, range of 1% to 20%, were found in the PI, NRTI and NNRTI. At the relaxed criteria, 17% of participants were in 8 clusters, ranging from 2 to 5 members. Those within clusters were less likely to be older, p=0.026 and more likely to be male, p=0029. Combination of current and prior dataset from the DO revealed 21 clusters, ranging from 2 to 6 members per cluster (Figure 2).

**Conclusion:**

In the DO, NNRTI-associated TDR is increasing, aligning with the continued use of Efavirenz as first-line ART highlighting the need for an update in the national guidelines. Identification of minority mutations suggests need for TDR surveillance. Detection and response to clusters is a novel, effective modality to understand HIV transmission networks and address care gaps at individual and systems level.

**Disclosures:**

**All Authors**: No reported disclosures

